# CO-CREATING EDUCATIONAL MATERIALS WITH OLDER ADULTS AND INFLUENCING HEALTH CARE DECISION-MAKERS

**DOI:** 10.1093/geroni/igae098.1743

**Published:** 2024-12-31

**Authors:** Wendy Hulko, Noeman Mirza

**Affiliations:** Thompson Rivers University, Kamloops, British Columbia, Canada; University of Winsdor, Toronto, Ontario, Canada

## Abstract

Rural and small city older adults’ views on primary and community care restructuring indicated a strong desire to provide input directly to healthcare decision-makers through oral over written means and collective rather than individual dialogues (Hulko et al., 2020). Thus, through integrated knowledge translation (iKT), we worked with older adults to (a) interpret our research findings on primary and community care restructuring; (b) create KT tools (grassroots infographic, key messages, documentary); and, (c) host a knowledge summit where we released our KT tools and facilitated discussions between service users and administrators (decision-makers, knowledge users, seniors advocates) about ways to move our findings into practice and affect service delivery. 30 people attended the knowledge summit and were invited to provide feedback on the iKT process and KT tools; 23 (8 service users, 15 administrators) filled out the feedback questionnaires. Service users provided positive feedback about the opportunity to partner with service providers, particularly senior administrators. Administrators recognized the need to shift from being “system focused” to “patient focused,” which can be facilitated through engagement of rural older adults in service delivery decisions. They recognized that service users had “good ideas on how to deliver services locally” and appreciated the opportunity to hear service users’ perspectives and needs, communicate face-to-face, and “discuss possible solutions” together. Our work highlights how emancipatory pedagogy - actioning research findings in collaboration with older adults - can enable mutually beneficial exchanges of ideas and solutions between service users and administrators and lead to improved health service delivery.

